# Clinical Outcomes Associated With the Addition of a Physical Therapist Assistant to a Rehabilitation Team When Treating Musculoskeletal Shoulder Pain in the Outpatient Setting: A Retrospective Cohort Study

**DOI:** 10.7759/cureus.42680

**Published:** 2023-07-30

**Authors:** Anthony N Baumann, Thad Indermuhle, Caleb J Oleson, Megan E Callaghan, Hudson Rogers, Caroline Pennacchio, Keith D Baldwin, John Martin Leland

**Affiliations:** 1 Department of Rehabilitation Services, University Hospitals, Cleveland, USA; 2 College of Medicine, Northeast Ohio Medical University, Rootstown, USA; 3 College of Medicine, Case Western Reserve University, Cleveland, USA; 4 Division of Orthopedics, Children's Hospital of Philadelphia, Philadelphia, USA; 5 Department of Orthopedic Surgery, University Hospitals, Cleveland, USA

**Keywords:** rehabilitation, orthopedics, treatment team, physical therapy assistant, physical therapy, shoulder pain

## Abstract

Introduction

Musculoskeletal shoulder pain (MSP) is a common condition frequently treated in an outpatient setting by a physical therapy rehabilitation team. Treatment teams can consist of physical therapists (PTs) with or without physical therapist assistants (PTAs). It is currently unknown how different physical therapy team compositions can impact patient outcomes in the outpatient setting. The purpose of this study is to examine how the addition of PTAs to a physical therapy treatment team would impact clinical outcomes when treating patients with MSP in the outpatient setting.

Methods

This study is a retrospective cohort analysis comparing clinical outcomes for pain, active range of motion (AROM), and disability for patients with MSP when treated by physical therapy treatment teams with or without the presence of PTAs. Inclusion criteria were patients treated for MSP in an outpatient physical therapy clinic without a history of shoulder surgery. Depending on the rehabilitation team composition, patients were divided into a PT-only group or a PTA group.

Results

Total patients (n = 238) had a mean age of 62.6 ± 12.6 years (median: 64 years) with a mean total number of physical therapy visits of 7.8 ± 4.9 visits (median: 7.0 visits). Of the entire cohort, the PT-only group had 100 patients and the PTA group had 138 patients. There was no significant difference in the magnitude of pain improvement (mean: 1.5 versus 1.9 points, p = 0.177), the magnitude of abduction AROM improvement (mean: 17.6 versus 13.9 degrees, p = 0.173), and the magnitude of disability improvement (mean: 18.9 versus 13.4 percentage points, p = 0.221) between the PT-only group and the PTA group. However, the PT-only group had significantly fewer total visits as compared to the PTA group (6.7 versus 8.6 visits, p < 0.001).

Conclusion

The addition of PTAs to a rehabilitation team when treating patients with MSP in the outpatient setting does not appear to adversely impact pain, AROM, or disability outcomes. However, patients treated only by PTs had significantly less visits with similar outcomes. More research is needed to determine the interplay between cost, healthcare utilization, and patient outcomes to maximize quality care.

## Introduction

Musculoskeletal shoulder pain (MSP) is a common disorder that can encompass many different shoulder pathologies, such as rotator cuff-related pain or adhesive capsulitis [1-5]. Physical therapy is a first-line conservative treatment option for MSP, with treatment usually occurring in outpatient physical therapy clinics and administered under the supervision of a rehabilitation team [1,4,6]. In the outpatient setting, physical therapy rehabilitation teams that treat MSP can consist of a physical therapist (PT) only or a PT along with a physical therapist assistant (PTA). The question as to the impact of using PTAs for physical therapy rehabilitation teams has been explored extensively in the literature in the acute and/or subacute setting in terms of patient outcomes, but very limited data exist for the outpatient setting [7-10]. Therefore, at this time, there exists no consensus as to the impact of physical therapy treatment team composition on patient outcomes, potentially limiting high-value care.

A similar question exists within other professions in the medical field via the utilization of advanced practice providers (APPs), such as physician assistants and nurse practitioners, in treatment teams led by physicians with numerous studies attempting to answer this question due to relevance to healthcare cost and patient outcomes [11-16]. However, the ideal composition of the physical therapy rehabilitation team in the outpatient setting when treating musculoskeletal conditions, such as MSP, remains unknown, as no studies to date have examined the impact of utilizing PTAs in the treatment of MSP in the outpatient setting. This question is especially relevant in the field of physical therapy at this time as recent changes in PT education levels and Medicare reimbursement continue to impact ideal rehabilitation team composition and cause significant shifts in the profession [7-9,17,18]. The purpose of this study is to examine the impact of physical therapy rehabilitation team composition via the use of PTAs on clinical outcomes, including pain, range of motion (ROM), and disability, when treating MSP in the outpatient setting to maximize healthcare utilization, decrease cost, and improve overall patient outcomes.

## Materials and methods

Study set up

This study is a retrospective cohort analysis comparing clinical outcomes for pain, ROM, and disability for patients with MSP when treated by physical therapy treatment teams with or without the presence of a PTA in the treatment team. All patients received conventional physical therapy (exercise, manual therapy, and physical modalities) as directed by the treating PT and no effort was made to control the interventions received by each patient to mimic real-world effects. This study was approved by the first author’s Institutional Review Board under study number #20220636. Patient charts were retrieved by the University Hospital’s Clinical Research Center for patients receiving physical therapy from 2016 to 2022 from multiple outpatient physical therapy clinics within a single hospital system.

Inclusion and exclusion criteria

Patients were included in this study if they were treated by a physical therapy team in an outpatient setting for MSP. Patients were also included if they had two or more physical therapy visits, i.e., continued physical therapy after the initial physical therapy evaluation. Patients were excluded if they had a history of shoulder surgery to remove a potential confounding factor for outcomes. Patients were excluded if they did not follow up after their initial PT evaluation, were not treated for MSP, or did not have pre and post-physical therapy data for pain.

Study definitions

For the purposes of this study, conventional physical therapy refers to the usual physical therapy care that a patient might receive in an outpatient physical therapy setting without any attempt by the authors to control the interventions given, thus simulating a real-world treatment environment. The “PT-only group” refers to patients who were treated by a physical therapy team that consisted only of PTs without the addition of PTAs. The “PTA group” refers to patients who were treated by a physical therapy team that consisted of PTs as well as PTAs. To belong to the PTA group, patients had to have at least one visit led by a PTA.

Data collection

Data were collected by multiple authors throughout the course of this study with any questions or conflicts settled by the first author. Data collected included patient age (years), male or female status, laterality of shoulder pain (left or right), the total number of visits, total number of visits completed by a PTA, pre and post-physical therapy pain outcomes (visual analog scale, VAS), abduction active ROM outcomes (degrees, measured by a goniometer), and disability outcomes (Quick DASH (Disabilities of the Arm, Shoulder, and Hand)). Patients who were treated for bilateral MSP were treated as two separate patient counts, with one data point for each shoulder, when complete data were available. From the total cohort of patients with complete pre and post-physical therapy pain outcomes, subgroup analysis was performed for ROM and disability outcomes due to incomplete data. For subgroup analysis, only patients with complete pre and post-ROM or disability outcomes were included in the data analysis.

Statistical analysis

This study utilized the Statistical Package for the Social Sciences (SPSS) version 29.0 (IBM Corp., Armonk, NY) software for statistical analysis. The Kolmogorov-Smirnov test or Shapiro-Wilk test was used to test the normality of the data based on sample size to determine the proper usage of parametric or non-parametric statistical analysis per outcome. Comparisons between two groups from independent samples were compared with the independent t-test or the Mann-Whitney U test depending on the nature of the data. Comparisons between two groups from the same sample, such as pre and post-physical therapy testing, were completed using the paired t-test or the Wilcoxon test. Descriptive statistics and frequencies were used to describe the demographic data. Statistical significance was set at p = 0.05 for all tests in this study.

## Results

Patient demographics

Included patients (n = 238) had a mean age of 62.6 ± 12.6 years (median: 64 years) with a mean total number of physical therapy visits of 7.8 ± 4.9 visits (median: 7.0 visits). Of the included patients, 41.2% of patients (n = 98) were male and 52.1% of the treated shoulders (n = 124) were right shoulders. Refer to Table 1 below for information on the demographics of the entire cohort. Based on treatment team composition, the PT-only group (n = 100) had a mean age of 61.5 ± 12.4 years (median: 63 years) with a mean total number of physical therapy visits of 6.7 ± 4.3 visits (median: 5.5 visits). The PTA group (n = 138) had a mean age of 63.3 ± 12.7 years (median: 64 years) with a mean total number of physical therapy visits of 8.6 ± 5.1 visits (median: 8.0 visits). The PTA-only group had a mean number of PTA visits of 4.3 ± 3.6 visits (median: 4.0 visits) per bout of physical therapy. There was no significant difference between the average age of both the PT-only group and the PTA group (p = 0.218). However, there was a statistically significant difference in the average number of total visits per group, with the PT-only group having significantly less visits as compared to the PTA group (p < 0.001). Refer to Table 2 below for information on the demographics of the included patients by group.

**Table 1:** Demographic information for all of the patients in the entire cohort for this study. Average represents mean ± standard deviation (median).

Demographic categories	Values
Entire cohort (n, %)	238 (100%)
Average age (years)	62.6 ± 12.6 (median: 64)
Average total physical therapy visits	7.8 ± 4.9 (median: 7)
Male (n, %)	98 (41.2%)
Female (n, %)	140 (58.8%)
Right shoulder (n, %)	124 (52.1%)
Left shoulder (n, %)	114 (47.9%)

**Table 2:** Demographic information for all of the included patients per group. Groups included the physical therapist (PT) only group as well as the physical therapy assistant (PTA) group. Average represents mean ± standard deviation (median).

Demographic categories	PT-only group	PTA group	Between-group p-values
Patients (n, %)	100 (42.0%)	138 (58.0%)	-
Average age (years)	61.5 ± 12.4 (median: 63)	63.3 ± 12.7 (median: 64)	p = 0.218
Average total physical therapy visits	6.7 ± 4.3 (median: 5.5)	8.6 ± 5.1 (median: 8)	p < 0.001
Average number of visits by PTA	-	4.3 ± 3.6 (median: 4)	-

Pain outcomes

Both the PT-only group and the PTA group had a significant improvement in pain from pre to post-physical therapy (p < 0.001). The magnitude of pain improvement in the PT-only group (n = 100) was 1.5 ± 2.6 points (median: 1.0 points). The mean pre-physical therapy pain score was 3.9 ± 3.1 points (median: 4.0 points) and the mean post-physical therapy pain score was 2.4 ± 2.8 points (median: 1.0 points) for the PT-only group. The magnitude of pain improvement in the PTA group (n = 138) was 1.9 ± 2.9 points (median: 2.0 points). The mean pre-physical therapy pain score was 5.0 ± 2.8 points (median: 5.0 points) and the mean post-physical therapy pain score was 3.1 ± 2.7 points (median: 2.5 points) for the PTA group. Unfortunately, there was a significant difference in the pre-physical therapy pain scores between groups with the PTA group having higher pain levels (p = 0.003). However, there was no significant difference in the magnitude of pain improvement between the PT-only group and the PTA group (p = 0.177). Refer to Table 3 for information on the magnitude of pain improvement within and between groups.

**Table 3:** Information on outcomes in terms of magnitude of improvement for pain, range of motion (ROM), and disability. Disability was defined via the Quick Disabilities of the Arm, Shoulder, and Hand (DASH) tool. Data are recorded for each of the three outcomes for both the physical therapist (PT) only group and the physical therapist assistant (PTA) group.

Outcome categories	PT-only group	PTA group	Between-group p-value
Magnitude of pain improvement (points)			
Mean ± standard deviation	1.5 ± 2.6	1.9 ± 2.9	p = 0.177
Median	1.0	2.0	
Patients (n, %)	100 (100%)	138 (100%)	
Magnitude of ROM improvement (degrees)			
Mean ± standard deviation	17.6 ± 26.7	13.9 ± 22.6	p = 0.173
Median	15.0	7.0	
Patients (n, %)	41 (41.0%)	59 (42.8%)	
Magnitude of disability improvement (percentage points)			
Mean ± standard deviation	18.9 ± 21.2	13.4 ± 17.2	p = 0.221
Median	13.2	12.0	
Patients (n, %)	23 (23.0%)	59 (42.8%)	

Range-of-motion outcomes

Both the PT-only group and the PTA group had a significant improvement in abduction ROM from pre to post-physical therapy (p < 0.001). The magnitude of ROM improvement in the PT-only group (n = 41) was 17.6 ± 26.7 degrees (median: 15.0 degrees). The mean pre-physical therapy ROM was 101.7 ± 45.5 degrees (median: 95.0 degrees) and the mean post-physical therapy ROM was 119.3 ± 44.0 degrees (median: 125.0 degrees). The magnitude of ROM improvement in the PTA group (n = 59) was 13.9 ± 22.6 degrees (median: 7.0 degrees). The mean pre-physical therapy ROM was 104.3 ± 35.5 degrees (median: 100.0 degrees) and the mean post-physical therapy ROM was 118.2 ± 32.4 degrees (median: 122.0 degrees) for the PTA group. There was no significant difference between the pre-physical therapy ROM between both groups (p = 0.749). There was no significant difference in the magnitude of ROM improvement between the PT-only group and the PTA group (p = 0.173). Refer to Table 3 for information on the magnitude of ROM improvement within and between each group.

Disability outcomes

Both the PT-only group and the PTA group had a significant improvement in disability measured via the Quick DASH from pre to post-physical therapy (p < 0.001). The magnitude of disability improvement in the PT-only group (n = 23) was 18.9 ± 21.2 percentage points (median: 13.2 percentage points). The mean pre-physical therapy disability score was 42.9 ± 24.7 percentage points (median: 45.0 percentage points) and the mean post-physical therapy disability score was 23.9 ± 20.0 percentage points (median: 22.0 percentage points) for the PT-only group. The magnitude of disability improvement in the PTA group (n = 59) was 13.4 ± 17.2 percentage points (median: 12.0 percentage points). The mean pre-physical therapy disability score was 47.7 ± 20.8 percentage points (median: 47.0 percentage points) and the mean post-physical therapy disability score was 34.3 ± 19.8 percentage points (median: 34.1 percentage points). There was no significant difference in pre-disability scores between both groups (p = 0.377). There was no significant difference in the magnitude of disability improvement between the PT-only group and the PTA group (p = 0.221). Refer to Table 3 for information on the magnitude of disability improvement within and between each group.

## Discussion

This study represents the first study to date to examine the impact of physical therapy rehabilitation team composition, via the presence or absence of PTAs, on clinical outcomes in the outpatient setting for patients with MSP. The attempt to determine the ideal treatment team composition is well attested in the literature in the medical field, either via APPs on physician-led teams or via the addition of PTAs to physical therapy rehabilitation teams in the acute and/or subacute settings [7-16]. However, until this study, no full manuscript to date examines the impact of using PTAs on a physical therapy rehabilitation team in the outpatient setting. Overall, the results of this study cautiously indicate that the addition of PTAs to a physical therapy rehabilitation team in the outpatient setting for the treatment of MSP does not result in any significant decrease in patient outcomes for pain, ROM, and disability.

Despite no significant decrease in outcomes, the question of cost remains as patients treated by only PTs had significantly less visits as compared to patients treated by a team consisting of PTs and PTAs. In a recent study examining the use of PTAs in an inpatient rehabilitation setting, higher PTA involvement in patient care after stroke did not significantly impact functional outcomes, length of stay in the inpatient rehabilitation facility, or change in discharge disposition [10]. Although these outcomes are different than the outcomes assessed in this study, the overall conclusion that PTAs did not adversely affect patient outcomes is consistent with the conclusions of this study [10]. Unfortunately, that study compared outcomes between treatment teams consisting of high or low PTA utilization, which is different than the grouping of this study, which included a group treated without PTAs at all [10]. Therefore, any comparisons between these two studies should be taken with caution, and more research is needed to determine the impact of the quantity of PTA utilization on patient outcomes. The current study did not examine how patient outcomes changed as PTA utilization increased within the PTA group. For example, it is unknown if patient outcomes in the outpatient setting would significantly change if 20% of patient visits were completed by a PTA versus 80% of patient visits. Overall, the literature has suggested that higher involvement of PTAs in the rehabilitation team could provide cost savings while maintaining patient outcomes [10]. Unfortunately, the results of this current study are limited in the recommendations for cost savings, and more research is needed.

It should also be noted that many factors impact ideal treatment team composition in the field of physical therapy. For example, a recent article examining PTA staffing and patient outcomes in skilled nursing facilities reported that higher physical therapy intensity, possibly improved by the utilization of PTAs, resulted in improved outcomes [8]. This resulted in a recommendation that skilled nursing facilities seeking to decrease cost while maintaining patient outcomes may choose to hire more PTAs rather than decrease physical therapy dosing and intensity [8]. However, as the outpatient setting is very different in terms of patient flow and scheduling, it is unknown if the same recommendations can be made from the available evidence of this study or extrapolated from subacute physical therapy studies to the outpatient setting at this time. Furthermore, the diagnoses treated by rehabilitation teams in the subacute setting, such as stroke, are vastly different than the diagnoses treated by rehabilitation teams in the outpatient setting [10]. Although no current full-text manuscript examines ideal rehabilitation team composition for other musculoskeletal conditions commonly seen in outpatient settings, preliminary data suggest the outcomes of this study may not be specific to MSP and may be consistent with other diagnoses, such as neck pain [19]. However, this fact is far from solidified, and more research is required.

At this time, this study has multiple limitations that impact the certainty of the results and limit application and generalizability. First, this study had a retrospective study design, thus likely introducing selection bias as to which patients were treated by teams consisting of only PTs versus teams consisting of PTs and PTAs. It is possible that PTs, who alone can perform the initial evaluation, could retain more complex patients in teams consisting only of PTs and only incorporate PTAs for patients who present with less complexity. This may be due to the gap in education levels between PTs and PTAs, although this relationship remains to be elucidated [17,18]. Furthermore, the selection bias could also be due to the different interventions available for the treatment of MSP, some of which fall outside of the skillset of PTAs, such as dry needling [20-24]. As this study did not control for the physical therapy interventions given to the patients within each group, the relative effectiveness of different interventions and the impact on patient outcomes could not be established with this study. However, the design of this study utilized conventional physical therapy without controlling for interventions as this mimics physical therapy treatment in the real world due to the heterogeneity of physical therapy interventions for MSP [1-3,5,23]. Furthermore, although the number of visits was specified, the amount of time spent with the patient per visit was not standardized or recorded, as this can vary between clinics within a hospital system. Another limitation is that some of the subgroup comparisons were not from equal subgroups, as the PTA group had significantly higher pain scores than the PT-only group. The impact of this fact is uncertain but could present a confounding factor for the results of this study. Finally, the relatively small sample size, especially for outcomes examined via subgroup analysis, could impact the significance of the results, and it is possible that larger sample sizes could show significance, although this fact is unknown at this time. The fact that all patients were treated within a single hospital system, although in multiple different outpatient clinics, limits the generalizability of the results of this study. Overall, this study serves as a foundation for future research on the impact of physical therapy rehabilitation team composition in the outpatient setting with the goal to improve healthcare utilization, decrease cost, and maximize patient outcomes.

## Conclusions

The addition of PTAs to a physical therapy rehabilitation team in the outpatient setting does not appear to significantly decrease pain, ROM, or disability outcomes in patients with MSP. However, patients treated only by PTs had significantly less visits with similar outcomes. This study is unable to comment on how these changes impact cost, thus requiring further research before recommending an ideal rehabilitation team composition in the outpatient setting. More research is needed to determine the interplay between cost, healthcare utilization, and patient outcomes to maximize quality care. Overall, this study cautiously suggests that the utilization of PTAs in the outpatient setting may not adversely impact patient outcomes for some musculoskeletal conditions, such as MSP, and adds to the conversation about ideal treatment team composition during physical therapy.
